# Irinotecan and vincristine for the treatment of refractory desmoplastic small round cell tumor in a developing country: a case report

**DOI:** 10.1186/s13256-019-1985-z

**Published:** 2019-03-10

**Authors:** Natalia Brenneken Duarte Ambar, Maria Teresa de Seixas Alves, Henrique Manoel Lederman, Simone Abib, Alexandre Alberto Barros Duarte, Eliana Monteiro Caran

**Affiliations:** Instituto de Oncologia Pediátrica – UNIFESP, Sao Paulo, Brazil

**Keywords:** Desmoplastic small round cell tumor (DSRCT), Irinotecan, Chemotherapy

## Abstract

**Introduction:**

Desmoplastic small round cell tumor is an extremely rare and aggressive cancer that affects mainly adolescents and young adults. Despite multiple therapeutic strategies, most patients have resistant disease with very poor survival rates.

**Case presentation:**

We present a case of a 10-year-old Caucasian boy with a desmoplastic small round cell tumor refractory to conventional treatment who exhibited a good response to alternative treatment. With use of irinotecan and vincristine in association with radiation therapy, a reduction of 96.9% of the dimensions of the target lesions compared with the initial image was observed.

**Conclusion:**

This chemotherapy regimen, in association with radiation therapy, demonstrated efficacy for refractory desmoplastic small round cell tumor in our patient, and it is cost-effective.

## Background

A desmoplastic small round cell tumor (DSRCT) is an extremely rare and highly aggressive cancer that affects adolescents and young adults. The tumor usually begins and spreads over the surface of the peritoneum [4, 12, 15, 16].

Over time, numerous approaches have been proposed for the treatment of DSRCT, and the best results have been obtained with the resection of more than 90% of the tumor, followed by the use of the Memorial Sloan Kettering Cancer Center P6 regimen, which uses vincristine, doxorubicin, and cyclophosphamide and alternates ifosfamide, mesna, and etoposide. However, this protocol induces high toxicity and a significant percentage of secondary treatment leukemia [13]. Despite multiple therapeutic strategies, most patients have a disease that is resistant to treatment, with various recurrences and very poor survival rates [3, 11, 14]. We report a case of a patient with DSRCT refractory to conventional treatment who exhibited a good response to alternative treatment with irinotecan, vincristine, and radiation therapy.

## Case presentation

Our patient was a 10-year-old Caucasian boy with abdominal pain and distention for 1 month before admission. He had no previous medical issues. Further examination showed tenderness in the lower quadrant of the abdomen with a palpable mass. We performed computed tomography (CT) of the abdomen, which identified an intraperitoneal mass in the hypogastrium, extending from the liver bottom edge and mesogastrium to the retrovesical space. A complete resection of the lesion was performed, and the histological results indicated DSRCT with the following immunohistochemical profile: positive for cytokeratin (AE1/AE3), EMA (Epithelial Membrane Antigen), Can5.2, 35bH11, CEA (Carcinoembryonic Antigen), desmin, WT1 (Wilms Tumor 1), synaptophysin, and enolase and negative for 34BE12, 1A4, CD99, and chromogranin. At that point, the patient was referred to our clinic for treatment.

Staging CT examinations of the chest showed a pulmonary nodule (7 mm) in the right lower lobe, and an abdominal CT showed that the tumor had spread into the abdominal cavity and liver nodules in segments V, VII, and VIII. According to the modified peritoneal cancer index, the patient’s cancer was classified as stage IV.

Treatment was started with the following chemotherapeutic regimen: vincristine (1.5 mg/m^2^ D1) + adriamycin (60 mg/m^2^ D1) + cyclophosphamide (1500 mg/m^2^ D1), alternating with ifosfamide (3 g/m^2^ D1–D3) + carboplatin (450 mg/m^2^ D1) + etoposide (150 mg/m^2^ D1–D3). After three cycles, a new abdominal CT scan showed stable disease, and the regimen was replaced by an alternative protocol with irinotecan (50 mg/m^2^ D1–D5) + vincristine (1.5 mg/m^2^ D1 and D7), resulting in an excellent partial response according to RECIST version 1.1 (Response Evaluation Criteria in Solid Tumors). After the 13th cycle, total abdominal radiotherapy was performed with a total dose of 2100 cGy, and subsequent irradiation of the areas with prior evidence of disease was performed, resulting in a total dose of 4440 cGy. A boost in the initial tumor bed (pelvis) and margin resulted in a total dose of 4980 cGy.

**Fig. 1:**
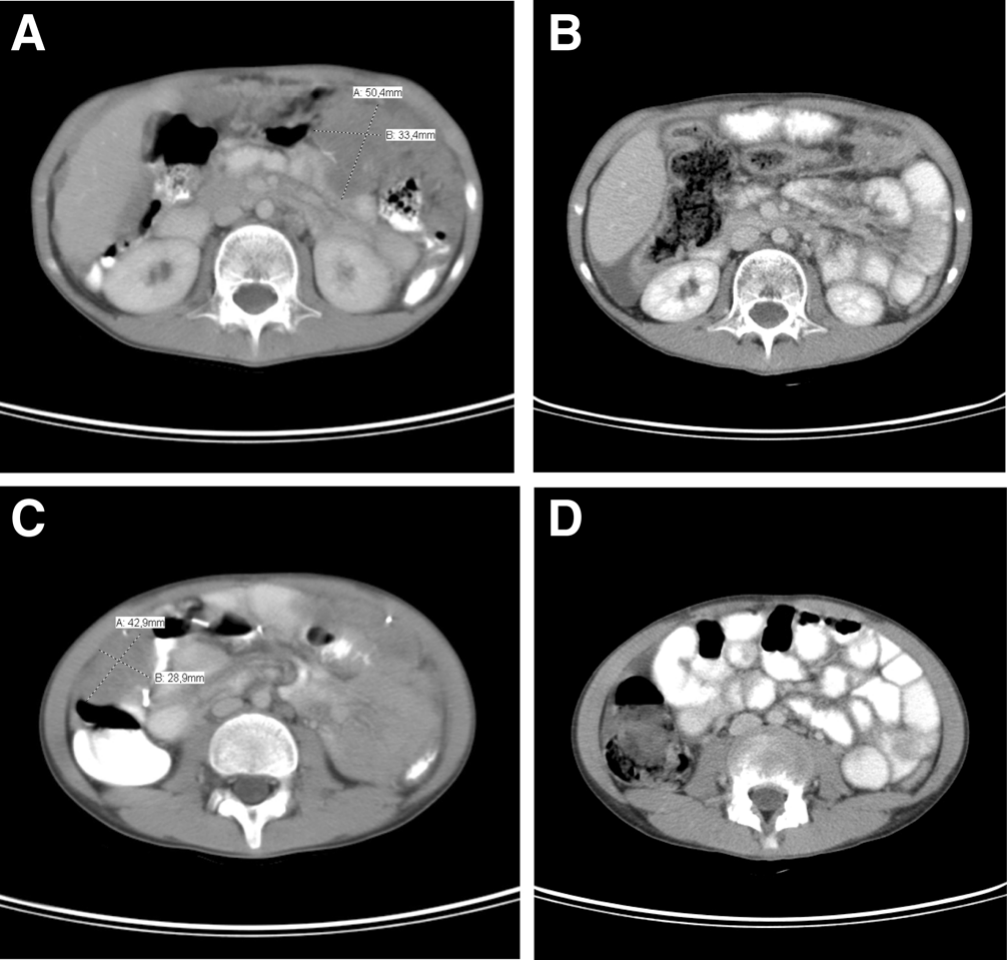
**a** Left kidney damage before treatment with irinotecan and vincristine. **b** Absence of injury after treatment. **c** Peri-cecal lesion prior to treatment with vincristine and irinotecan. **d** Absence of injury after treatment

After initiation of treatment, the first reassessment (after four cycles) showed a 36.7% reduction compared with the initial image, without the appearance of new lesions. In the second and third reevaluations (after 10 and 16 cycles, respectively), reductions of 47.7% and 87.7%, respectively, were observed. At the last CT evaluation, a reduction of 96.9% of the tumor in relation to the original image was observed, without the appearance of new lesions, resulting in a notable partial response (Fig. 1).

After 21 cycles, maintenance chemotherapy was initiated with oral cyclophosphamide (25 mg/m^2^/day) and vinorelbine (D1, D8, and D15; 25 mg/m^2^/week). Fifteen cycles of maintenance chemotherapy were conducted, and a positron emission tomography/CT evaluation after the treatment showed no anomalous hypermetabolism. During the treatment, the patient had episodes of grade 2 diarrhea, which were treated with loperamide without any severe adverse events.

After 46 months from the diagnosis, the patient was admitted to our hospital with an acute obstructed abdomen. After imaging examinations and a laparotomy, tumor recurrence was diagnosed. The patient died at 50 months after diagnosis.

## Discussion and conclusions

Knowledge of the desmoplastic tumor has accumulated since it was described in 1989. Aggressive behavior with local and distal spread has frequently been reported in the literature. Currently, the immunohistochemical diagnosis of this tumor is well established, as well as the specific chromosomal translocation t(11;22)(p13;q12) *EWS* gene juxtaposed with the tumor suppressor gene *WT1* [1, 8]. Yet, despite the advances and new isolated therapies with complex treatment plans, the prognosis of patients with desmoplastic tumors remains poor, especially in cases with distant metastases, and new therapeutic options need to be studied [10].

Unless the tumor is an incidental finding, most patients have advanced abdominal disease with large masses, visceral dissemination, and deployment of parietal peritoneum preventing a radical surgical approach. In the literature, the presence of metastases at diagnosis is most commonly observed in the liver, lung, and lymph nodes [9, 10]. In this context, effective neoadjuvant chemotherapy facilitates systemic control, and surgery facilitates local control. In most studies, the therapeutic regimen includes the chemotherapy protocol of the Ewing family, with an emphasis on alkylating agents, which are effective for small cell tumors. However, the therapeutic responses obtained with this scheme are often insufficient, and the toxicity is high.

The first description of the treatment of a desmoplastic tumor using irinotecan dates to 1999 in a publication in which Rosoff *et al.* [13] reported the use of isolated irinotecan with clinical improvement of one of the two patients evaluated, but the study did not include the patients’ survival time. In 2007, Hayes-Jordan *et al.* [5] used irinotecan associated with temozolomide as the first-line treatment for a patient with an event-free survival (EFS) of 10 months, and this therapy was used as a second-line treatment for a patient with an EFS of 6 months. After 2 years, in 2009, Bisogno *et al.* [2] again used isolated irinotecan as a second-line treatment for eight patients, which resulted in stable disease in some patients and a maximum overall survival (OS) of 19 months. The association of vincristine with this scheme was then suggested.

In 2013, Hirano *et al.* [8] also published data using isolated irinotecan as second-line treatment for patients with EFS and OS of 6 and 20 months, respectively. The following year, Fan *et al.* [4]. again tested the combination of temozolomide with irinotecan as a second-line treatment for two patients, resulting in EFS of 6 and 15 months.

With the experience gained in the use of irinotecan chemotherapy alone or in combination with temozolomide for the treatment of a desmoplastic tumor, we performed a new alternative regimen with vincristine, irinotecan, radiation therapy, and maintenance chemotherapy for our patient as a salvage treatment and obtained a partial response of over 95%, with EFS and OS comparable to those in the literature.

The difference in the cost of medications should be evaluated. For example, in Brazil, the cost of one 100-mg tablet of temozolomide costs about $144.00, and the cost of one 1-mg ampoule of vincristine costs $3.40. The use of temozolomide makes the treatment 40 times more expensive, and this cost difference should be considered in countries with limited resources.

With this chemotherapy regimen associated with radiation therapy, a reduction of 96.9% of the dimensions of the target lesions compared with the initial image was observed, resulting in EFS and OS of 26 and 50 months, respectively. Taking in account the results of Hayes-Jordan *et al.* [6, 7], our patient would likely have benefited from surgery and hyperthermic intraperitoneal chemotherapy, but the technique was not available in our service at that time.

The chemotherapy regimen of vincristine, irinotecan, radiation therapy, and maintenance chemotherapy demonstrated efficacy for refractory DSRCT in our patient, and it is cost-effective, suggesting the possibility of its incorporation as a first-line treatment, especially in countries with limited resources. Although we achieved a satisfactory cost-benefit balance with this regimen, it is important to emphasize that it is a rare tumor and that we had only one patient, which makes a comprehensive analysis more difficult. Therefore, in order to make this regimen useful in clinical practice, new prospective, multicenter research with an adequate number of patients is required.
